# Evaluation of Rapid Library Preparation Protocols for Whole Genome Sequencing Based Outbreak Investigation

**DOI:** 10.3389/fpubh.2019.00241

**Published:** 2019-08-27

**Authors:** Helena M. B. Seth-Smith, Ferdinando Bonfiglio, Aline Cuénod, Josiane Reist, Adrian Egli, Daniel Wüthrich

**Affiliations:** ^1^Division of Clinical Bacteriology and Mycology, University Hospital Basel, Basel, Switzerland; ^2^Applied Microbiology Research, Department of Biomedicine, University of Basel, Basel, Switzerland; ^3^DBM Bioinformatics Core Facility, SIB Swiss Institute of Bioinformatics, Basel, Switzerland; ^4^Personalized Health Basel, University of Basel, Basel, Switzerland

**Keywords:** NGS, next generation sequencing, library, Illumina, whole genome sequencing, comparison, bacteria, prokaryotes

## Abstract

Whole genome sequencing (WGS) has become the new gold standard for bacterial outbreak investigation, due to the high resolution available for typing. While sequencing is currently predominantly performed on Illumina devices, the preceding library preparation can be performed using various protocols. Enzymatic fragmentation library preparation protocols are fast, have minimal hands-on time, and work with small quantities of DNA. The aim of our study was to compare three library preparation protocols for molecular typing: Nextera XT (Illumina); Nextera Flex (Illumina); and QIAseq FX (Qiagen). We selected 12 ATCC strains from human Gram-positive and Gram-negative pathogens with %G+C-content ranging from 27% (*Fusobacterium nucleatum*) to 73% (*Micrococcus luteus*), each having a high quality complete genome assembly available, to allow in-depth analysis of the resulting Illumina sequence data quality. Additionally, we selected isolates from previously analyzed cases of vancomycin-resistant *Enterococcus faecium* (VRE) (*n* = 7) and a local outbreak of *Klebsiella aerogenes* (*n* = 5). The number of protocol steps and time required were compared, in order to test the suitability for routine laboratory work. Data analyses were performed with standard tools commonly used in outbreak situations: Ridom SeqSphere+ for cgMLST; CLC genomics workbench for SNP analysis; and open source programs. Nextera Flex and QIAseq FX were found to be less sensitive than Nextera XT to variable %G+C-content, resulting in an almost uniform distribution of read-depth. Therefore, low coverage regions are reduced to a minimum resulting in a more complete representation of the genome. Thus, with these two protocols, more alleles were detected in the cgMLST analysis, producing a higher resolution of closely related isolates. Furthermore, they result in a more complete representation of accessory genes. In particular, the high data quality and relative simplicity of the workflow of Nextera Flex stood out in this comparison. This thorough comparison within an ISO/IEC 17025 accredited environment will be of interest to those aiming to optimize their clinical microbiological genome sequencing.

## Introduction

Whole genome sequences currently provide the highest resolution for typing bacterial pathogens. The implementation of next generation sequencing (NGS) in routine clinical microbiology laboratories provides the foundation to analyze bacteria with high resolution, reproducibility and accuracy. Decreasing costs and increasing ease of implementation through increasingly flexible platform options, means that more laboratories will seek this technology over time.

Whole genome sequencing (WGS) has shown its value in molecular epidemiology, from seminal papers on MRSA and *Mycobacterium tuberculosis* helping to trace and resolve epidemics (1, 2), to implementation in routine laboratories (3–5), and local molecular epidemiological studies (6, 7). Methods of analysis range from determination of multi-locus sequence type (MLST; low resolution) through core genome MLST (cgMLST; high resolution) to whole genome phylogenies based on single nucleotide polymorphisms (SNPs; highest resolution). Using WGS in outbreak detection ideally takes account of all mutations and genomic variability in order to fully resolve outbreak scenarios and transmission chains (5, 8–11). Factors encoded within the genomes, such as antimicrobial resistance (AMR) and virulence factors, can also be determined from good quality assemblies (3–5, 12). Quality assurance, backward compatibility, communication between experts in different fields, and reporting to clinicians are issues currently being addressed (13–17).

Behind all these analyzes lies the all-important data. Several technologies have been used over the past decade for WGS: Ion Torrent PGM, Roche 454, PacBio and most recently Oxford Nanopore Technologies. But it is predominantly data from Illumina machines, from the MiniSeq, MiSeq, NextSeq, or HiSeq platforms, that is used for molecular epidemiology or bacterial genomics, as evidenced by the vast amounts of Illumina data deposited in databases (>90% at the Short Read Archive). Prior to the sequencing step, DNA libraries need to be made, protocols for which can vary greatly. Given the relatively high cost of library preparation compared to sequencing, and the time required to perform it, library preparation is a critical and rate-limiting step. Although many aspects of WGS can be optimized for routine diagnostic microbiology (17), to date few studies have addressed the data quality produced by different library methods.

Mechanical shearing of DNA often offers the most even and controllable DNA fragmentation (18), but requires high amounts of input DNA and hands-on time. Automation of mechanical shearing is problematic, limiting throughput. The most popular and implementable library protocols use proprietary transposases to cleave the DNA and ligate the adapters in one step, a method which is rapid but dependent on the DNA/enzyme concentration ratio, and is subject to sequence bias. The impact of this bias on the %G+C rich *Mycobacterium tuberculosis* genome has been explored, and the TruSeq (Illumina) method, involving mechanical shearing of DNA, was found to be superior to the enzymatic Nextera XT (Illumina) (19). On the AT-rich *Plasmodium falciparum* genome, Nextera was again found to give highly biased results (20). This phenomenon has also been observed in human leukocyte antigen (HLA) genotyping (21).

With QIAseq FX, Qiagen have recently released a library preparation protocol that is based on fully enzymatic fragmentation (nuclease). The advantage of this approach is that the efficiency of the fragmentation is not as strongly affected by %G+C-content as the transposase from the Nextera XT approach. As QIAseq FX uses only an enzyme and not a whole complex, the adaptor ligation must then be applied in a separate step (QIAseq FX DNA Library Handbook). Another recent launch, Nextera Flex (Illumina) is also a transposome based library preparation kit, promising consistent yield and fragment size, and less sequence bias (22). The development over Nextera XT involves bead-conjugated transposomes, meaning that the tagmentation sites are positionally better defined by the DNA binding to the beads.

The costs of the different compared kits are quite similar, and up-to-date prices are listed on the manufacturer's websites. Currently, the difference across all is <20%. Some laboratories implement protocols using lower reagent volumes to reduce the per sample costs, however this study used the manufacturer's standard protocols.

Our aim was to compare the data quality from three commercial library preparation kits, for use in clinical routine microbiology WGS. The optimal protocol is rapid, performs consistently across all genome types without optimization, and produces high quality data for both rapid and reliable outbreak analysis and AMR gene detection.

## Materials and Methods

### Strain Selection

In order to evaluate the usability of the different library preparation kits, we made a selection of 12 ATCC strains representing Gram-positive and negative pathogenic bacterial species, with a high range of %G+C-content (Table 1). A complete high-quality reference genome exists for each strain. Additionally, we included seven local patient isolates of *Enterococcus faecium* and five isolates from a *Klebsiella aerogenes* outbreak from 2018.

**Table 1:** List of sequenced isolates, characteristics, reference genomes, and sample accessions.

**Unique name**	**Species**	**DNA extraction conczentration (ng/μl)**	**Reference used**	**%G+C- content reference**	**Reference sequence accession**	**Number of reads produced**			**Sample accession**
						**XT**	**Flex**	**Qia**	
ATCC25586	*Fusobacterium nucleatum*	36.4	ATCC25586	27.15	NC_003454.1	1,16,59,182	54,71,621	38,92,304	ERS3207828 (SAMEA5402510)
ATCC700819	*Campylobacter jejuni*	34.2	ATCC700819	30.55	NC_002163.1	51,04,723	80,67,749	5,37,051	ERS3207833 (SAMEA5402515)
ATCC25923	*Staphylococcus aureus*	88.4	ATCC25923	32.86	NZ_CP009361.1, NZ_CP009362.1	90,93,138	57,42,025	71,39,563	ERS3207824 (SAMEA5402506)
ATCC29212	*Enterococcus faecalis*	39.8	ATCC29212	37.35	NZ_CP008816.1, NZ_CP008815.1, NZ_CP008814.1	71,99,132	68,06,105	69,81,047	ERS3207826 (SAMEA5402508)
ATCC19615	*Streptococcus pyogenes*	20.8	ATCC19615	38.48	NZ_CP008926.1	78,95,584	60,46,735	94,81,835	ERS3207823 (SAMEA5402505)
ATCC25845	*Prevotella melaninogenica*	92.0	ATCC25845	40.98	NC_014370.1, NC_014371.1	69,93,760	21,62,813	52,57,867	ERS3207831 (SAMEA5402513)
ATCC25922	*Escherichia coli*	27.2	ATCC25922	50.37	CP009072.1	64,19,681	53,21,879	61,12,711	ERS3207827 (SAMEA5402509)
ATCC700603	*Klebsiella quasipneumoniae*	42.8	ATCC700603	57.73	NZ_CP014696.2, NZ_CP014697.2, NZ_CP014698.2	48,25,887	58,53,388	84,17,937	ERS3207829 (SAMEA5402511)
ATCC25177 (H37Ra)	*Mycobacterium tuberculosis*	1.2	ATCC25177	65.61	NC_009525.1	47,94,204	96,95,720	2,54,69,645	ERS3207832 (SAMEA5402514)
ATCC27853	*Pseudomonas aeruginosa*	42.4	ATCC27853	66.08	CP015117.1	45,32,729	48,88,025	68,12,269	ERS3207825 (SAMEA5402507)
ATCCBAA-67	*Burkholderia stabilis*	72.0	ATCCBAA-67	66.42	NZ_CP016442.1, NZ_CP016443.1, NZ_CP016444.1	87,99,551	55,77,758	63,87,296	ERS3207822 (SAMEA5402504)
ATCC4698	*Micrococcus luteus*	45.6	ATCC4698	73.00	CP001628.1	50,81,588	93,96,130	85,84,319	ERS3207830 (SAMEA5402512)
NMB004374	*Enterococcus faecium*	55.8	Aus0004	37.80	NC_017022.1	53,87,832	52,12,078	77,60,492	ERS3207811 (SAMEA5402493)
NMB004375	*Enterococcus faecium*	55.8	Aus0004	37.80	NC_017022.1	52,85,502	44,62,856	55,05,430	ERS3207812 (SAMEA5402494)
NMB004376	*Enterococcus faecium*	55.4	Aus0004	37.80	NC_017022.1	49,36,762	28,48,407	88,145	ERS3207813 (SAMEA5402495)
NMB003061	*Enterococcus faecium*	56.2	Aus0004	37.80	NC_017022.1	41,98,651	52,13,009	84,72,370	ERS3207814 (SAMEA5402496)
NMB003076	*Enterococcus faecium*	47.2	Aus0004	37.80	NC_017022.1	61,97,648	64,57,841	75,28,868	ERS3207815 (SAMEA5402497)
NMB003240	*Enterococcus faecium*	57.6	Aus0004	37.80	NC_017022.1	71,97,873	75,30,750	81,14,687	ERS3207816 (SAMEA5402498)
NMB003062 (VRECH001)	*Enterococcus faecium*	40.6	Aus0004	37.80	NC_017022.1	51,80,248	77,99,226	76,15,044	ERS2595418 (SAMEA4775467)
NMB004427	*Klebsiella aerogenes*	38.6	KCTC2190	55.00	NC_015663.1	62,65,626	34,05,502	68,37,549	ERS3207817 (SAMEA5402499)
NMB004428	*Klebsiella aerogenes*	25	KCTC2190	55.00	NC_015663.1	9,64,566	78,95,179	1,09,31,859	ERS3207818 (SAMEA5402500)
NMB004429	*Klebsiella aerogenes*	29	KCTC2190	55.00	NC_015663.1	4,27,144	67,01,537	60,70,575	ERS3207819 (SAMEA5402501)
NMB004430	*Klebsiella aerogenes*	24.8	KCTC2190	55.00	NC_015663.1	33,39,975	38,92,829	69,09,301	ERS3207820 (SAMEA5402502)
NMB004431	*Klebsiella aerogenes*	28.2	KCTC2190	55.00	NC_015663.1	46,70,831	86,19,114	56,84,285	ERS3207821 (SAMEA5402503)

### DNA Extraction and Sequencing

All work was performed in an ISO/IEC 17025 accredited environment, although only the Nextera XT protocol is currently accredited. DNA from all isolates was extracted by Qiagen EZ1 (Qiagen, Hilden, Germany) using the DNeasy blood and tissue kit (Qiagen), from a single colony. Prior to this, some isolate were subject to pretreatment: *Mycobacterium tuberculosis* was inactivated at 95°C for 1 h and disrupted in a TissueLyser (Qiagen) for 2 min at highest frequency; *Streptococcus pyogenes* was pre-treated with the TissueLyser (Qiagen) for 2 min at frequency 30; *Staphylococcus* were pre-treated with lysozyme und lysostaphin for 30 min at 37°C; all other bacteria were pre-treated using Proteinase K for 10 min at 56°C. Extracts were quantified by Qubit (Invitrogen), separated into three aliquots, and frozen at 20°C.

Libraries were created from the aliquots using Nextera XT (“XT”; Illumina), Nextera DNA Flex (“flex”; Illumina) or QIAseq FX (“Qia”; Qiagen). The recommended amounts and concentrations of DNA for each protocol were used where possible (1 ng for XT, 100 ng for Flex, 200 ng for Qia). To simulate a more realistic situation for *M. tuberculosis*, for which DNA extraction is not trivial, we used less DNA for the three kits (1 ng for XT, 10 ng for flex, 10 ng for Qia).

Each pool of libraries was loaded and sequenced separately on a NextSeq 500 device (cluster densities: XT 202, flex 189, Qia 244 K/mm^2^) and were sequenced using 2 × 151 bp paired end reads, within the Division of Clinical Microbiology, University Hospital Basel. The data was demultiplexed using bcl2fastq (version v2.17.1.14; Illumina).

### Genomic Data Quality Analysis

Reads were trimmed using trimmomatic (version 0.38) (23) using default parameters (ILLUMINACLIP:2:30:10 SLIDINGWINDOW:4:15 MINLEN:125), and randomly subsampled using seqtk (version 1.3-r106, -s100; https://github.com/lh3/seqtk) to provide mean 10, 20, 50, 100, and 200-fold coverage of the genomes.

Assemblies were produced by unicycler (v0.3.0b) (24), with assembly parameters derived using QUAST (version 5.0.2) (25). The annotation was performed using Prokka (version 1.13) (26). AMR genes were predicted by using ABRicate (version 0.8.10; https://github.com/tseemann/abricate) with the NCBI database (accession: PRJNA313047).

Reads from ATCC strains were mapped using BWA (version 0.7.17) (27) against the complete references with all replicons concatenated (Table 1). The read depth at the different positions was determined using pilon (version 1.23). The insert size was calculated from the sam files using an in-house python script (https://github.com/danielwuethrich87/collection/blob/master/scripts/parse_sam_for_insertsize.py). The base-composition at the difference positions within the reads was calculated using FastQC (version 0.11.5; https://www.bioinformatics.babraham.ac.uk/projects/fastqc/) on the mapped 10-fold subsampled reads from ATCC25586.

K-mer signatures of sub-sampled reads and corresponding reference genomes were computed with Sourmash v2.0.0 (28) using the suggested MinHash resolution (1000:1 compression ratio) and a k-mer size of 31. The k-mer signature of the subsampled assemblies was assessed with the Jaccard distance metric, which is calculated by asking how many k-mers are shared between two samples vs. how many k-mers in total are in the combined samples [(Sample1 ∩ Sample2)/(Sample1 ∪ Sample2)]. A Jaccard distance of 1 means the samples are identical; a Jaccard distance of 0 means the samples are completely different. Overlaps with reference genome were also calculated in terms of containment [(Sample1 ∩ Sample2)/Sample1].

The orthologous groups were determined using the stand-alone Roary pipeline v3.12.0 (29), which takes annotated assemblies in GFF3 format produced by Prokka as above. Principal component analysis (PCA) was performed on the output table of gene presence/absence and the coordinates of the first two principal components (weighted by the proportion of variance explained) were used to calculate the distance of each sample from the reference as a metric to determine the similarity in terms of gene content.

### Outbreak Analysis

For outbreak isolate genomes, data was analyzed in Ridom SeqSphere+ v4.1.6 for *Enterococcus faecium* cgMLST (30), and *Klebsiella aerogenes* cgMLST using an *ad-hoc* scheme comprising 3282 target loci based on the KCTC2190 genome (NC_015663.1) and 41 additional genomes from NCBI. Additionally, MentaLiST (version 1.0.0) (31) was used to identify the cgMLST alleles from the *Enterococcus faecium* isolates.

CLC Genomics Workbench 10.1 was used to generate Single Nucleotide Polymorphism (SNP) phylogenies. Mapping was performed using default parameters, variant calling used the parameters: 10x min coverage, 10 min count and 70% min frequency. SNP trees used a neighbor joining method: minimum coverage 10, minimum coverage 10%, minimum z-score 1.96, multi-nucleotide variants included. The mapping reference for the *Klebsiella aerogenes* outbreak was that of KCTC2190, accession number CP002824.

The *Enterococcus faecium* data was also analyzed using snippy (version 4.3.6, –minfrac 0.8; https://github.com/tseemann/snippy) for SNP calling comparing to the Aus0004 as reference (accession number NC_017022.1) For the phylogenetic analysis, only the core genome SNPs were used. The phylogenetic tree was calculated using the neighbor joining tree algorithm of the scikit-bio (version 0.2.0) package (http://scikit-bio.org/).

## Results

### Library Preparation and Ease of Use in Routine Laboratories

We selected three different rapid library preparation kits, all of which are based on enzymatic fragmentation: Nextera XT (“XT”), Nextera DNA Flex (“flex”), and QIAseq FX DNA (“Qia”) as they each provide a complete solution kit. The required DNA input amount of the three kits is very variable: XT needs exactly 1 ng of input DNA; Qia and flex support a wide range of DNA inputs that can affect the library preparation. The insert size of the Qia kit can be controlled by adjusting the fragmentation time and DNA input amount (1–1,000 ng). Flex accepts a wide range of input DNA (1–500 ng) resulting in the same insert size (300–350 bp). However, in Qia and flex, different DNA input amounts require the number of cycles in the PCR amplification step to be adjusted. Qia also supports a PCR free protocol if more than 100 ng are applied. We decided to use 1 ng of input DNA for XT, 100 ng for flex and 200 ng for Qia. This amount of DNA is reliably produced by our routine DNA extraction techniques, and simplifies the Qia protocol through elimination of PCR. For *M. tuberculosis* we used only 10 ng for Qia and flex. For the Qia protocol we were aiming for a fragment peak size of 550 bp by using 6 min fragmentation time for 100 ng and 10 min for the 10 ng input. The other kits do not allow specific adjustments for fragment length in the standard protocol. Each of the final libraries using XT and Qia were quantified and the 24 samples were equimolarly pooled. As sample normalization is already included in flex, we pooled the samples by taking the same volume from each library.

The application of the three kits revealed their strengths and weaknesses in the laboratory. For routine work, time is of course a major factor. The provider of all three kits state that the library preparation takes 2.5 h. However, we were only able to reach this time with XT, and only if time taken for DNA quantification before and after is not included. We also have to mention that the XT protocol has been established in our laboratory for 3 years and therefore the technicians are highly experienced. The Qia and flex protocols both took ~4 h. The Qia kit requires long fragmentation time (~60 min) and ligation time (45 min). It also has to be considered that if the input DNA amount of Qia is below 100 ng, PCR and clean-up must be included, which adds a further 90 min. For flex, the resuspension of the beads with the transposomes requires optimization, as they stick to the walls of PCR plates. Saving hands-on time, especially with larger sample numbers, flex includes bead-based concentration normalization. Also of importance in routine laboratory work, the Illumina kits provide plenty of consumable, which allows for potential inaccurate pipetting and still allows the indicated number of samples to be processed. In contrast, the Qia fragmentation mixture volume delivered in the kit was too limited and resulted in the sequencing failure of one sample (NMB004375).

Taken together, XT has the most convenient protocol to use in the laboratory. However, flex provides some features that allow a very streamlined process. Even though the flex protocol takes longer than XT, the wide range of DNA input amount and the normalized output can lead to a significant time gain. The Qia protocol take also longer than the XT protocol and needs more adjustments according to the DNA input amounts. On the other hand, it offers the easy adjustment of insert sizes.

### Genome Coverage Evaluation of ATCC Strains

**Figure 1:**
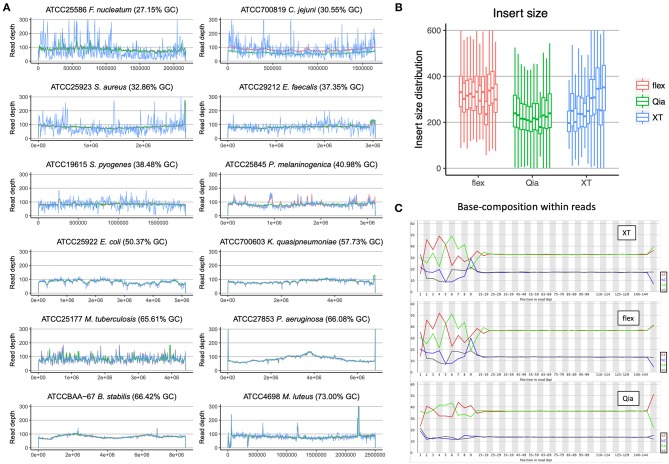
Quality assessment of WGS data. **(A)** The reads of the three library kits subsampled to 100-fold were mapped against the 12 reference genomes and the read depth called was measured. The colors indicate the different library preparation kits. The x-axis reflects the position along the genomes and the y-axis the read depth. **(B)** The insert size of the different libraries was calculated using the alignment of the paired-end reads to the reference. The boxplots represent the calculations from the different species, with the lowest %G+C-content on the left, and the highest on the right. In the boxplots the lower and upper hinges correspond to the first and third quartiles. The whiskers are located at 1.5x of the interquartile range. **(C)** The base composition of all the nucleotide sites in the reads was determined. The bases on the left side show the composition around the fragmentation site.

As a first estimate for the quality of the sequencing we mapped the reads of the ATCC strains against their published reference genomes. For this purpose, we aligned the 100-fold subsampled reads from each sample to the reference and visualized the read depth distribution (Figure 1A). The read depth is most variable using XT. This is especially obvious in genomes with low %G+C-content, resulting in many genomic regions with low coverage. Qia and flex, on the other hand, show a more even distribution of read depth in all samples and therefore provide a more complete representation of the genomes. The unevenness of coverage is less pronounced in genomes with G+C-content of 40% or more: these show similar pictures with XT, flex and Qia.

Based on the alignments of the reads to the reference genomes we calculated the insert sizes of the different library preparation kits (Figure 1B). With XT we see a clear trend that the genomes with higher %G+C-content have larger insert sizes, showing again that this method is highly sensitive to %G+C-content. The insert sizes of the flex and Qia are stable across the different genomes, with the exception of the low input *Mycobacterium tuberculosis*, and seem unaffected by %G+C-content. Using flex, the insert sizes are well above 300 bp, which allows an optimal use of 151 × 151 paired-end reads. With Qia we have an insert size slightly above 200 bp, despite having aimed for 400 bp (550 bp fragment size). This value should be able to be adjusted through in-depth protocol optimization.

Looking at %G+C-content variation within the reads (Figure 1C), overall the reads produced by Qia and flex are closer to the actual genomic %G+C-content than those from XT. Focusing on the beginning of the reads, which represent the fragmentation sites, flex and XT give a strong variation of the %G+C-content, which is characteristic for the transposome used by Nextera. Surprisingly this fragmentation preference does not affect the read depth distribution of flex. Using Qia we see that the beginning of the reads are very similar to the %G+C-content of the genomes.

### Evaluation of Assembly Quality

**Figure 2:**
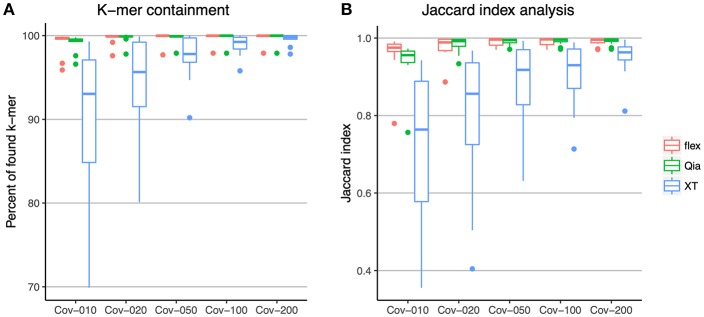
Comparison of the sequencing content using k-mers. **(A)** All k-mers identified within the reads were compared to those k-mer from the reference genomes. The x-axis shows the different subsampling of the reads and the y-axis shows the percent of k-mers that were found in the reads. **(B)** The assemblies of the sequenced strains were compared against the reference assemblies using the Jaccard index of the k-mers. The x-axis shows the different subsampling of the reads used for each assembly. The y-axis shows the Jaccard index. The colors indicate the different library preparation kits. In the boxplots the lower and upper hinges correspond to the first and third quartiles. The whiskers are located at 1.5x of the interquartile range.

In order to study the genome representation in the different library preparation kits, we analyzed the subsampled reads of the ATCC strains at mean 10, 20, 50, 100, and 200-fold coverage. K-mer containment was used to compare the k-mers in the reads of the difference subsamples against the k-mers in the reference assemblies (Figure 2A). With this analysis we found that, using Qia and flex with an average read depth of 10-fold, more than 99% of all k-mers were found in most of the genomes. At 50-fold with these two kits, k-mers were already completely saturated, indicating that all the genome is represented. XT shows a different picture: while increasing read depth increases the percentage of k-mers found, the k-mer pool of the reference is not completely represented using XT even with 100 and 200-fold coverage, indicating that there will always be regions absent, leading to incomplete representation of the genome.

*De novo* assembly of the subsampled reads was performed, and the k-mers of the assemblies compared to those from the references using the Jaccard index (Figure 2B). This analysis shows a similar picture. Qia and flex show a good representation of the genome with a 50-fold coverage upwards, whereas using XT with 100-fold and 200-fold coverage, reads do not completely represent the genome.

Assembly quality measures (NG50, number of contigs, genome representation, mismatches) were calculated using Quast (Figure S1). With increasing coverage, contig length (NG50) increases, as does genome fraction compared against the reference genomes, the number of contigs in the assembly decreases, and so do the number of mismatches called between the assemblies and the references. This analysis again shows that we can obtain an almost complete representation of the genome with 50-fold coverage using Qia and flex; XT on the other hand needs 100-fold or more coverage. In order to compare the gene content of assemblies from the different library preparation kits and subsamples, we performed a PCA on the presence and absence of orthologous groups (Figure S2). In general, we found that low coverage assemblies (10- and 20-fold) are more likely to result in different gene content (less genes) to the references, which cluster with the high coverage assemblies. However, we also found that in strains with low %G+C-content (≤50.37%; ATCC25586, ATCC700819, ATCC25923, ATCC29212, ATCC19615, ATCC25845, and ATCC25922) the genes found in the XT assemblies, even at high coverage, are separated from the references, Qia and flex assemblies.

AMR genes were analyzed in the published complete ATCC reference genomes and the assemblies from our experiment, using ABRicate (Table 2). We found that we can find every resistance gene from flex and Qia reads if the coverage is 50-fold or over. With XT many genes are not found with a coverage of 50-fold and some genes are even absent from the assemblies produced from a coverage of 100 or 200-fold.

**Table 2:** Prediction of AMR determinants in sequenced ATCC strains compared to reference genomes.

**ATCC strain**	Resistance mechanism^*^			**Automatically detected in**														
				**XT**					**Flex**					**Qia**				
	**Name**	**% coverage**	**% identity**	**10**	**20**	**50**	**100**	**200**	**10**	**20**	**50**	**100**	**200**	**10**	**20**	**50**	**100**	**200**
ATCC25177	*aac(2')-Ic*	100	100	Y	Y	Y	Y	N	Y	N	Y	Y	Y	Y	Y	Y	Y	Y
	*erm(37)*	100	100	Y	Y	Y	Y	Y	Y	Y	Y	Y	Y	N	N	Y	Y	Y
	*blaA*	100	100	Y	Y	Y	Y	Y	Y	Y	Y	Y	Y	Y	Y	Y	Y	Y
ATCC25922	*blaEC-5*	100	100	Y	Y	Y	Y	Y	Y	Y	Y	Y	Y	Y	Y	Y	Y	Y
ATCC25923	*tet(38)*	100	100	2	2	2	2	Y	N	Y	Y	Y	Y	Y	Y	Y	Y	Y
	*fosD*	100	79.05	N	N	N^**^	N	Y	Y	Y	Y	Y	Y	Y	Y	Y	Y	Y
ATCC27853	*fosA*	100	98.53	Y	Y	Y	Y	Y	Y	Y	Y	Y	Y	Y	Y	Y	Y	Y
	*catB7*	100	99.22	Y	Y	Y	Y	Y	Y	Y	Y	Y	Y	Y	Y	Y	Y	Y
	*blaOXA-396*	100	100	Y	Y	Y	Y	Y	Y	Y	Y	Y	Y	Y	Y	Y	Y	Y
	*aph(3')-IIb*	100	98.39	Y	Y	Y	Y	Y	Y	Y	Y	Y	Y	Y	Y	Y	Y	Y
	*blaPDC-303*	100	99.92	P	Y	Y	Y	Y	Y	Y	Y	Y	Y	Y	Y	Y	Y	Y
ATCC29212	*dfrE*	100	97.98	Y	Y	Y	Y	Y	Y	Y	Y	Y	Y	P	Y	Y	Y	Y
	*tet(M)*	100	100	Y	Y	Y	Y	Y	P	Y	Y	Y	Y	Y	Y	Y	Y	Y
	*lsa(A)*	100	99.8	Y	Y	Y	Y	Y	Y	Y	Y	Y	Y	Y	Y	Y	Y	Y
ATCC700603	*blaOKP-B-23*	100	99.42	Y	Y	Y	Y	Y	Y	Y	Y	Y	Y	Y	Y	Y	Y	Y
	*oqxA10*	100	93.79	Y	Y	Y	Y	Y	Y	Y	Y	Y	Y	Y	Y	Y	Y	Y
	*oqxB11*	100	95.94	Y	Y	Y	Y	Y	Y	Y	Y	Y	Y	Y	Y	Y	Y	Y
	*fosA*	100	95.24	P	Y	Y	Y	Y	Y	Y	Y	Y	Y	Y	Y	Y	Y	Y
	*blaSHV-18*	100	100	Y	Y	Y	Y	Y	Y	Y	Y	Y	Y	Y	Y	Y	Y	Y
	*ant(2”)-Ia*	100	100	Y	Y	Y	Y	Y	N	Y	Y	Y	Y	Y	Y	Y	Y	Y
	*aphA16*	100	100	N	N	2	2	Y	N	Y	Y	Y	Y	2	Y	Y	Y	Y
	*aadA10*	100	87.59	N	Y	Y	Y	Y	N	Y	Y	Y	Y	Y	Y	Y	Y	Y
	*blaOXA-2*	100	100	N	Y	Y	Y	Y	N	Y	Y	Y	Y	Y	Y	Y	Y	Y
	*qacEdelta1*	100	100	N	Y	Y	Y	Y	N	Y	Y	Y	Y	Y	Y	Y	Y	Y
	*sul1*	100	100	N	Y	Y	Y	Y	N	Y	Y	Y	Y	Y	Y	Y	Y	Y
ATCC700819	*blaOXA-605*	99.75	99.63	P	N	N	Y	Y	Y	Y	Y	Y	Y	Y	Y	Y	Y	Y
ATCCBAA-67	*penA*	95.74	84.89	Y	Y	Y	Y	Y	Y	Y	Y	Y	Y	Y	Y	Y	Y	Y

* *All under 70% coverage and/or 70% identity were screened out. Y, identified; N, not identified (red); P, partial (yellow); 2, split over 2 contigs (yellow)*.

** *This sequence also assembled a contig of 896 bp which is predicted to carry a dfrC resistance determinant: % coverage 91; % identity 76*.

### Estimation of Coverage Required for cgMLST Analysis

In 2018, we sequenced, as routine, several cases of vancomycin-resistant *Enterococcus faecium* (VRE) and a small outbreak of *Klebsiella aerogenes* (*K. aerogenes*) that was not associated with our hospital. For this study, we selected five *K. aerogenes* isolates and seven VRE isolates to evaluate the performance of the three kits on samples from the routine clinical microbiology laboratory.

**Figure 3:**
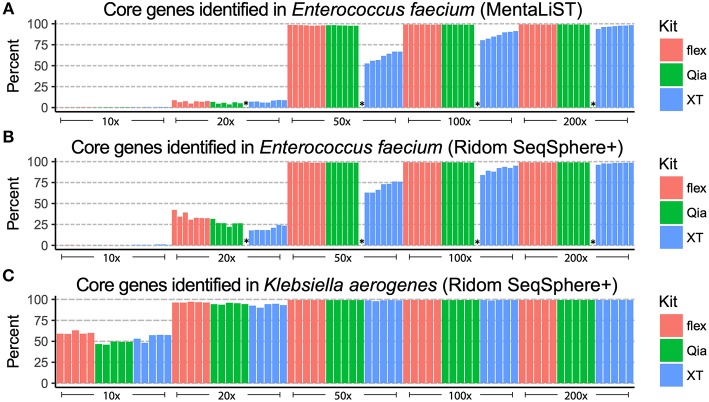
cgMLST alleles identified from the patient isolates. The different subsamples (x-axis) were used to determine of the alleles of the core genome. The different strains are depicted as bars. The y-axis shows the percentage of core genes that can be used for allelic typing. The colors indicate the different library preparation kits. The *E. faecium* isolates were analyzed using Mentalist **(A)** and Ridom SeqSphere+ **(B)**. The *K. aerogenes* isolates were analyzed only using Ridom SeqSphere+ **(C)**. The failed Qia library is labeled with “*”.

After subsampling, we typed the seven VRE strains using the cgMLST scheme of *Enterococcus faecium*, in the commercial software Ridom SeqSphere+, and using the open source software MentaLiST. In order to determine the resolution, we compared the number of core genes found in each sample and subsample (Figures 3A,B). Using reads from Qia and flex libraries, most of the core genes are found with a 50-fold coverage and over. With XT, over 25% of the core genes are not identified using a coverage of 50-fold, and 10–20% are still missing at 100-fold coverage.

For the five *K. aerogenes* we created an *ad-hoc* cgMLST scheme using Ridom SeqSphere+. In comparison to the VRE, 50-fold coverage was sufficient for all three kits to assign alleles to over 85% of core genes (Figure 3C). As *K. aerogenes* has a higher %G+C-content than *E. faecium*, we have seen that this results in more equal genome coverage from all kits, especially XT, leading to better assemblies and increased core gene identification.

### Analysis of Vancomycin-Resistant *Enterococcus faecium* Isolates

A previous investigation showed an outbreak of VRE from Switzerland carried the same MLST type (ST796) as an outbreak in Australia (32–34). Out of this investigation we selected four isolates from an outbreak, as well as three isolates (ST117) from an example of in-patient acquisition of a vancomycin-resistance carrying transposon (Tn1549) in the same strain background. To test the performance of the different kits we aligned the reads against a reference (Aus0004) for the construction of a SNP scheme. For the analysis we only selected SNPs from the core genome to reduce false SNPs caused by the distance to the reference. Using high coverage samples (≥50-fold Qia, 50-fold flex, 100-fold XT), we found more than 2,000 SNPs between the isolates of ST117 and ST796. In contrast, within each sequence type only 1-2 SNP differences were identified (Figure S3A). All strains showed the same distance to the reference at the root of the tree (Figure S3B). However, if we also include samples with lower sequencing depth (≤20-fold Qia, ≤20-fold flex, ≤50-fold XT), we find a higher diversity in the pairwise comparison of the strains (Figure S3C): up to 49 SNPs among ST117 isolates, and in up to 51 SNPs among the ST796 isolates. This is an indication that we are discovering falsely called SNPs. The neighbor joining phylogeny also shows that that subsamples with lower sequencing depth have a smaller distance to the root, as not all SNPs are called (Figure S3D). Therefore, we conclude that we can improve SNP typing: lowering the number of falsely called SNPs and increasing the number of “real” SNPs, by using higher sequencing coverage, and Qia and flex kits.

We identified AMR genes in the VRE isolates (Table S1). Using Qia and flex, all AMR genes are found if at least a coverage of 50-fold is used. For XT, a coverage of at least 100-fold is needed to ensure the detection of all genes.

### Analysis of *Klebsiella aerogenes* Outbreak Analysis

**Figure 4:**
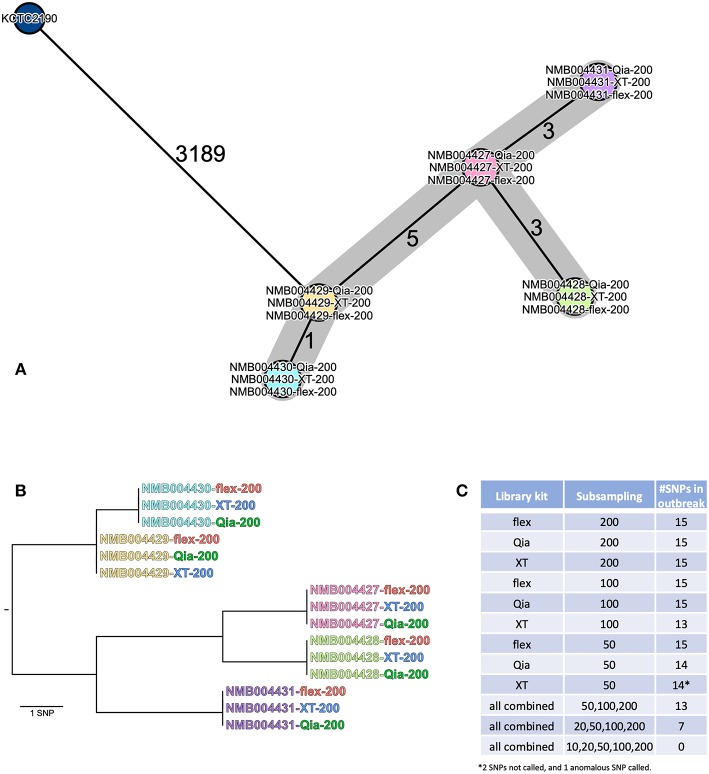
Analysis of the *K. aerogenes* outbreak isolates. **(A)** The isolates (200-fold subsamples) were analyzed using cgMLST in Ridom SeqSphere+ and are depicted in a minimum spanning tree (MST). The isolates are shown as circles. If two strains are identical they collapse into one circle. The numbers on the lines connecting the different circles show the number of different alleles between two isolates (not to scale). **(B)** The genomic distances between the isolates (200-fold subsamples) is show as a phylogenetic tree representing all SNP differences across the whole genome. **(C)** SNP numbers across the tree called using the different subsamples.

Five *K. aerogenes* isolates from a small outbreak were investigated using the commercial software Ridom SeqSphere+ for cgMLST, and CLC genomics for SNP analysis. The cgMLST analysis gave the same results for the all library kits at 200-fold (Figure 4A), showing small numbers of allelic discriminations between the isolates. Using lower coverage subsampled datasets, the number of identified allelic differences becomes smaller (Figure S4). Using flex, all the strains could be differentiated even with 10-fold coverage, which was not the case for Qia and XT, where isolates began to collapse into clusters. In the SNP analysis we did not find any differences between the kits with 200-fold coverage (Figure 4B), with each identifying 15 SNPs separating the five isolates. However, if we perform the analysis with the lower subsampled reads, again the resolution declines (Figure 4C). We could still capture the whole diversity using flex with 100-fold and 50-fold coverage, and Qia with 100-fold. Using XT we identified 13 and 14 SNPs (1 SNP was falsely called) in the 100-fold and 50-fold dataset, respectively.

## Discussion

### Sequence Quality

This in-depth comparison of three commercial library preparations kits shows the superiority of the Qia and flex kits over XT concerning the quality of the data produced. Through our strategic study design, including a range of human pathogens, we have shown that these two methods produce high quality NGS data that represent the whole genome, at a mean coverage of at least 50-fold. The fragmentation step of these methods is highly stable to variability in the %G+C-content in the genome, resulting in almost even distribution of read depth. In contrast, XT is highly affected by the %G+C-content variation within and between the genomes. This results in an incomplete representation of the genome, especially if lower read depths (<100-fold) are used. Therefore, we suggest that, while Qia and flex libraries can be relied on at mean coverages 50-fold and above, a higher sequencing depth for libraries prepared with XT is required (over 100-fold), which will affect the number of samples that can be pooled on a sequencing run (Table S2). This is crucial for the highest resolution of typing, and for comprehensive surveillance of genomic elements such as AMR and virulence genes. We note that we found limitations of the XT data in terms of genome representation in some cases even at a mean read depth of 200-fold.

Our study protocol used a single DNA extraction protocol, and as such we cannot exclude that the tested kits show a different performance with other protocols. Additionally, we conducted this study without technical replicates, therefore variability between batches could not be assessed.

### Outbreak Investigation

The investigations of the *E. faecium* and *K. aerogenes* patient isolates show the strength of WGS for bacterial typing. Even though the three kits are based on significantly different enzymatic and chemical reactions, the typing results are identical between the methods at high coverage. If the data quality is low, resolution is lost, both in cgMLST and SNP analysis. This is a very important finding for typing laboratories, and especially large-scale projects that want to compile NGS data from nationwide labs to establish national surveillance (35). In all settings, however: local, national or global, the quality control and bioinformatic analysis remain key for epidemiological analysis, as low-quality data can affect the outcomes by lowering the resolution or allowing the false calling of SNPs.

### Usability in the Laboratory

The evaluation of the usability in the laboratory showed that XT is the quickest protocol (2.5 h). The core protocols of Qia and flex take a least 1 h longer. However, in the flex protocol, library normalization is included, which reduces the time needed to pool the libraries. This protocol also offers a flexible input amount (50–500 ng) that does not require optimization, saving time in the DNA preparation. However, if <50 ng is available, the number of PCR cycles has to be increased. The Qia protocol needs an accurate measurement of the input DNA, as the resulting fragmentation depends on the DNA amount. If <100 ng input DNA is used, additional PCR and clean-up steps are required that prolong the library preparation by a further 60–90 min. Therefore, XT is superior in time efficiency, but closely followed by flex.

The fragment length is also a very important factor in the sequencing process. Libraries with insert sizes that are smaller than the read length lead to overlaps in the reads pair and therefore loss of sequence information. Fragment length also affects the cluster density calculation, the clustering efficiency and the sequencing depth. If the fragment length is stable across different sample types, the amount of DNA is sufficient data to calculate the molarity and therefore the number of clusters. Long fragments lead to inefficiency in the clustering and can result in very low cluster densities; short fragments can lead to over clustering and the failure of a run. Therefore, it is important to produce a stable insert size for the libraries, independent of the input DNA. Our comparisons showed that the insert length in Qia and flex are stable across varying %G+C-content. The insert size from the XT is much more affected by the %G+C-content. In our experience with XT, we obtain much higher cluster densities (occasionally leading to over clustering) when sequencing AT-rich species such as *Campylobacter*, as opposed to *Klebsiella*. It is worth mentioning that we suggest to use 2 × 150 bp reagent kits for these libraries, as the tested libraries generally show an insert size of <350 bp. We do not recommend using the MiSeq Reagent Kit v3 (600-cycle) for these libraries, as it produces 2 × 300 bp reads, and the resulting read pairs would overlap with libraries of this length.

Current trends indicate that WGS will be used more often in routine diagnostics and therefore also the number of samples processed will increase. Thus, an implementation of the library preparations kits on automated liquid handling systems will reduce the time and cost associated with this technology. All three protocols discussed in this study can be implemented on liquid handling systems that are equipped with a thermocycler and a magnetic stand.

We have summarized the important features of the different kits that should support other labs in deciding on the most appropriate library preparation kit (Table 3).

**Table 3:** Key features of the compared library preparation kits.

	**Nextera XT**	**Nextera DNA Flex**	**QIAseq FX**
Time required	2.5 h	4 h	4 h
DNA input amount range (ng)	1–1	1–500	1–1,000
Adjustments required for variable input	No variable input supported	PCR cycles required to be adjusted, using <50 ng	Additional PCR step is required if using <100 ng (+ 90 min)
Insert size behavior	Affected by DNA input amount and %G+C-content	Barely affected by the input DNA	Affected by DNA input amount
Available barcodes	384	384	96
Limitations	Highly affected by input DNA	Bead-linked transposomes (BLT) handling needs practice	Reagent volumes are tight
Key advantage	Simple protocol	Highly standardized output (input DNA independent)	PCR-free (>100 ng input DNA)
Special feature	Fast protocol	Produces normalized libraries (>100 ng input DNA)	Insert size can easily be adjusted to needs
Data quality	Highly variable read depth	High quality data	High quality data
Recommended read depth	G+C < 50%: 200 x G+C ≥ 50%: 100 x	50 x	50 x

## Final Conclusion

The evaluation of the three kits clearly showed that the data quality from libraries made with Qia and flex are superior to those from XT. The comparison of laboratory processes of the Qia and flex kits shows that flex is superior, as the protocol needs very few adjustments, and less hands-on time for routine questions. Therefore, flex best enables streamlining of the laboratory processes for WGS in the context of surveillance.

## Data Availability

The datasets generated for this study can be found in the ENA repository under project PRJEB31421.

## Author Contributions

HS-S, FB, and DW performed the data analysis and wrote the manuscript. AC and JR performed the data analysis. AE wrote the manuscript.

### Conflict of Interest Statement

The authors declare that the research was conducted in the absence of any commercial or financial relationships that could be construed as a potential conflict of interest.
