# Supplementary material for: Evaluation of Rapid Library Preparation Protocols for Whole Genome Sequencing Based Outbreak Investigation
Source: Front Public Health. 2019 Aug 27;7:241. doi: 10.3389/fpubh.2019.00241 (PMC6719548; doi:10.3389/fpubh.2019.00241)
Supplement: Supplementary file 1 [file Image_1.pdf]

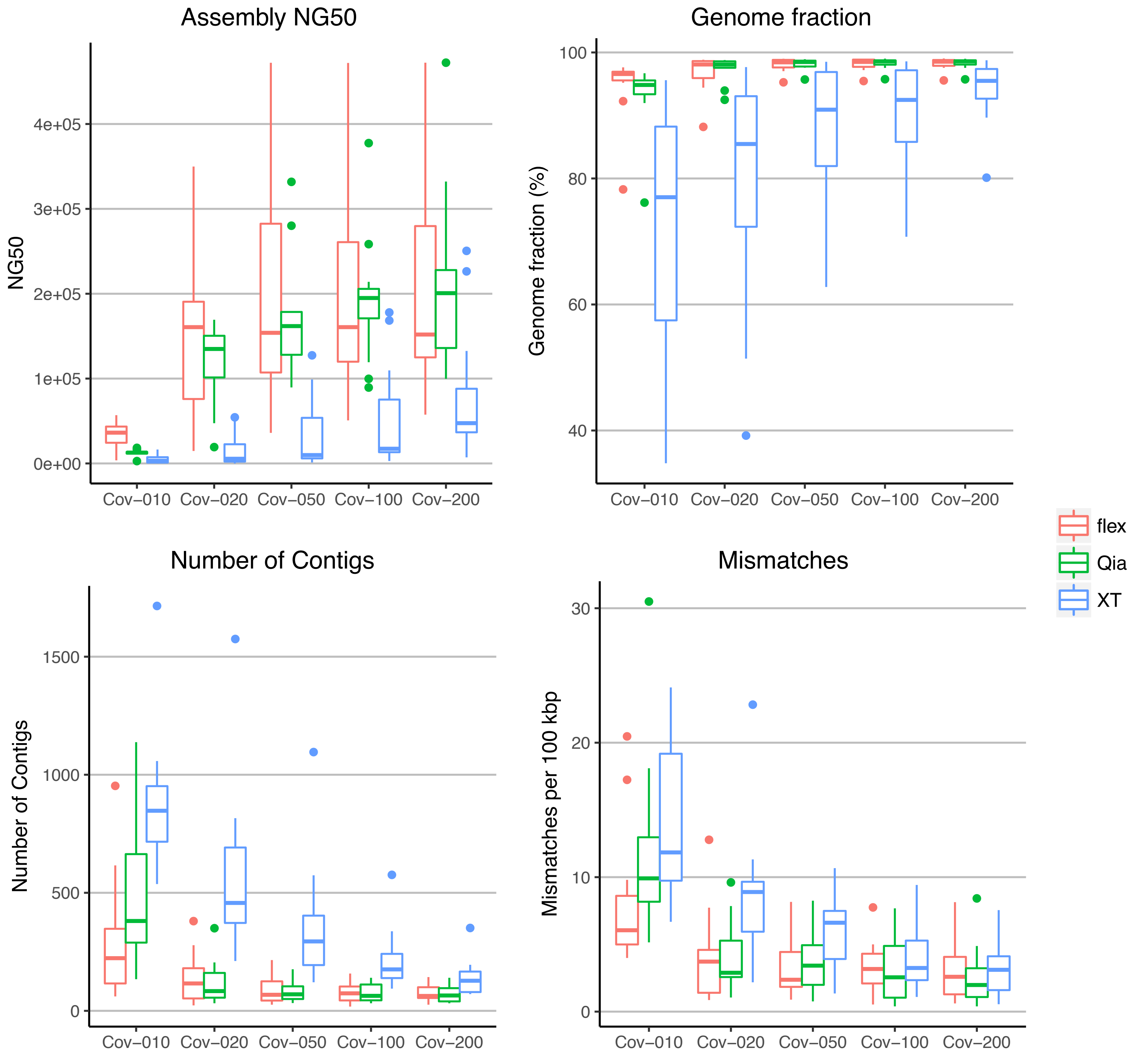

Figure S1: Quality measures of the *de novo* assemblies. The different subsamples of the reads were *de novo* assembled. The assembly quality was assessed by calculating four important quality measures. The subsampling is indicated on the x-axis and the measure on the y-axis. The colors indicate the library kit used. In the boxplots the lower and upper hinges correspond to the first and third quartiles. The whisker are located at 1.5x of the interquartile range.
