# Supplementary material for: Evaluation of Rapid Library Preparation Protocols for Whole Genome Sequencing Based Outbreak Investigation
Source: Front Public Health. 2019 Aug 27;7:241. doi: 10.3389/fpubh.2019.00241 (PMC6719548; doi:10.3389/fpubh.2019.00241)
Supplement: Supplementary file 2 [file Image_2.pdf]

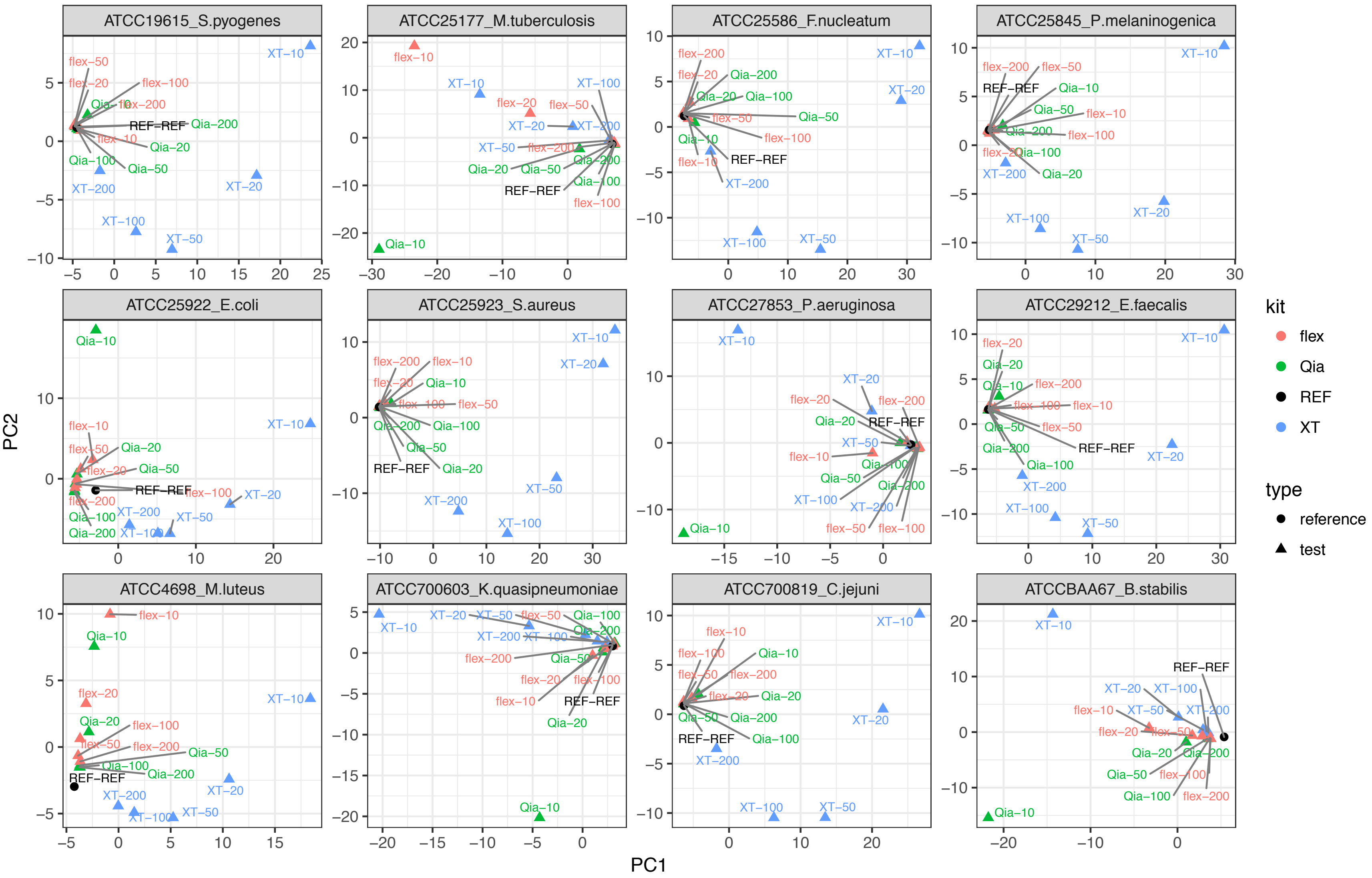

Figure S2: PCA analysis of the orthologous groups. The orthologous groups of the different assemblies from subsampled reads were built using Roary. A PCA was performed to define whether an orthologous gene cluster is present or absent in an assembly. The different strains analysed are depicted in individual boxes. The kit and subsampling is indicated in the figure. The x-axis shows principal component one the y-axis principal component two. Points close to the reference are those with similar orthologous gene cluster in the assemblies.
