# Supplementary material for: Evaluation of Rapid Library Preparation Protocols for Whole Genome Sequencing Based Outbreak Investigation
Source: Front Public Health. 2019 Aug 27;7:241. doi: 10.3389/fpubh.2019.00241 (PMC6719548; doi:10.3389/fpubh.2019.00241)
Supplement: Supplementary file 3 [file Image_3.pdf]

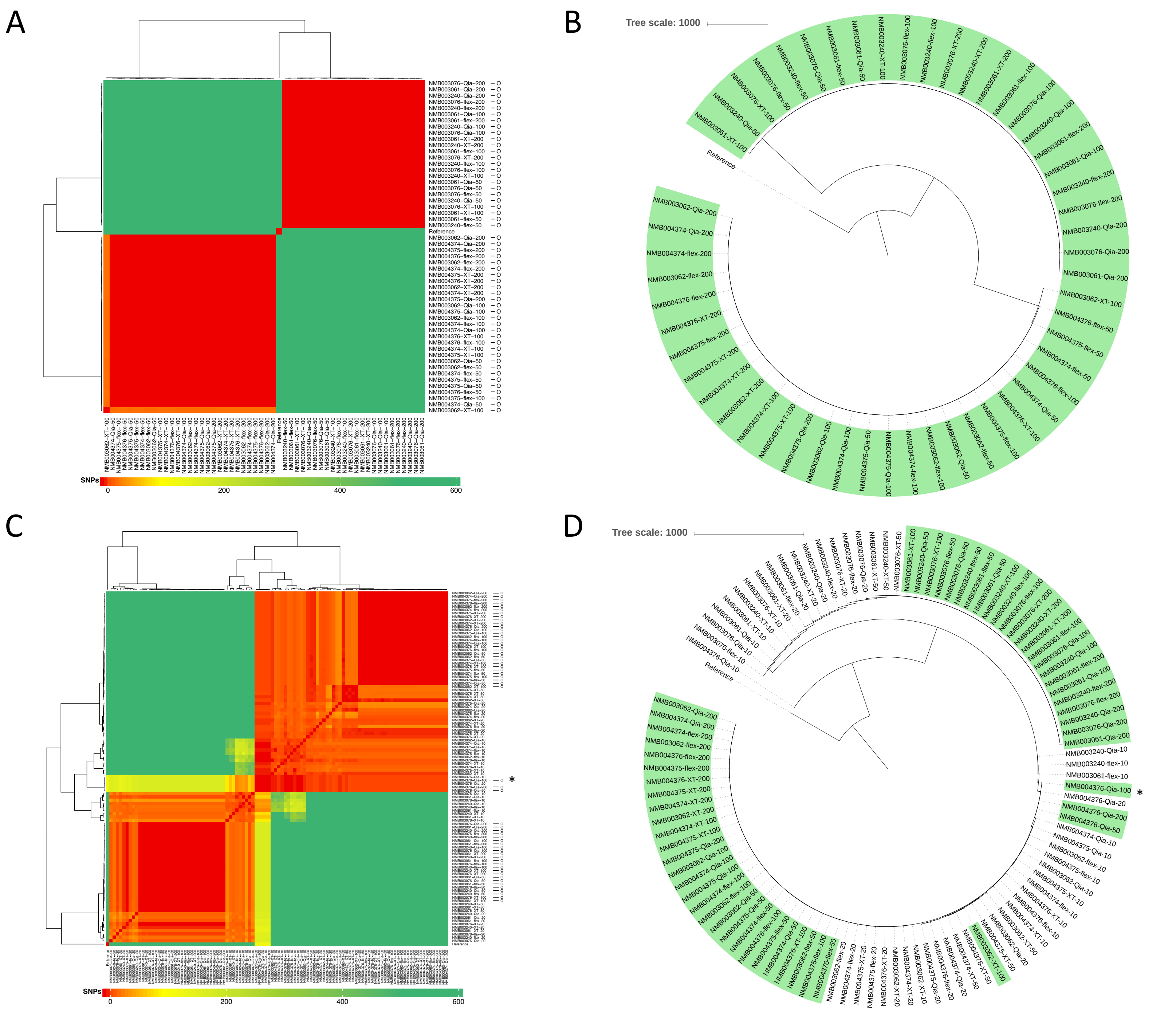

Figure S3: Core genome based phylogenetic analysis of the *E. faecium* isolates. A and **B** only included high quality libraries: Qia and flex with 50 x coverage or more and XT with at least 100 x**. C** and **D** include all sequenced samples and their subsamples. **A** and **C** are heatmaps showing the pairwise SNP comparison of the core genomes of the strains. The color scale indicates the number of SNPs identified between two strains. High quality libraries are labeled with a "-O". **B** and **D** show the neighbor joining trees of the isolates based on core genome SNPs. High quality libraries are marked in green. Two clusters of highly similar isolates are apparent, with lower coverage libraries showing increased numbers of SNPs. The failed Qia library is labeled with "*".
