# Supplementary material for: Evaluation of Rapid Library Preparation Protocols for Whole Genome Sequencing Based Outbreak Investigation
Source: Front Public Health. 2019 Aug 27;7:241. doi: 10.3389/fpubh.2019.00241 (PMC6719548; doi:10.3389/fpubh.2019.00241)

Figure S4. Comparison of Ridom SeqSphere+ generated cgMLST minimum spanning trees (MSTs) by library kit and coverage. Isolates are colored for orientation. Reference isolate KCTC2190 is shown in dark blue. Minimum distances between isolates is shown with lines, and the number of allele differences is superimposed on the lines. This is not shown to scale. Complexes (≤20 allele differences as defined in Ridom SeqSphere+) are highlighted in grey. The resolution between the isolates and the precise configuration of the MST changes across kits, especially at lower coverage, but the definition of the complex remains constant.

XT-200

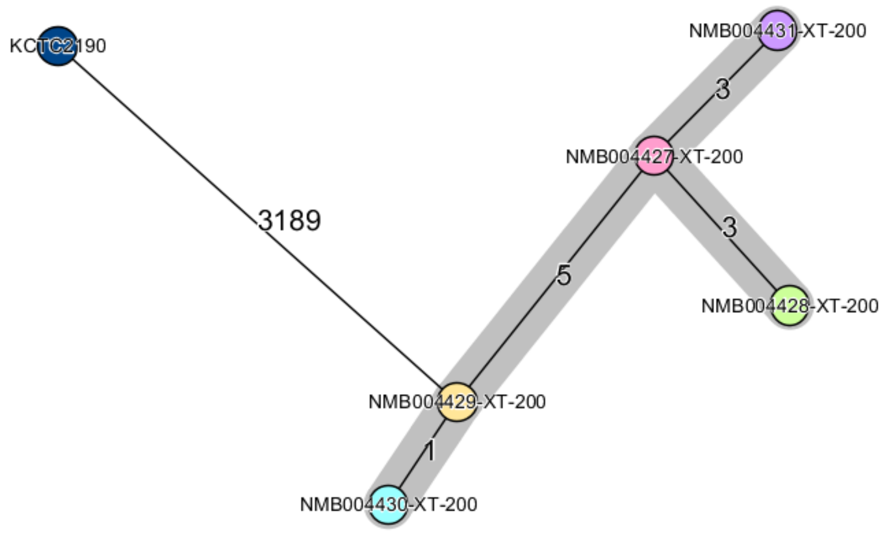

flex-200

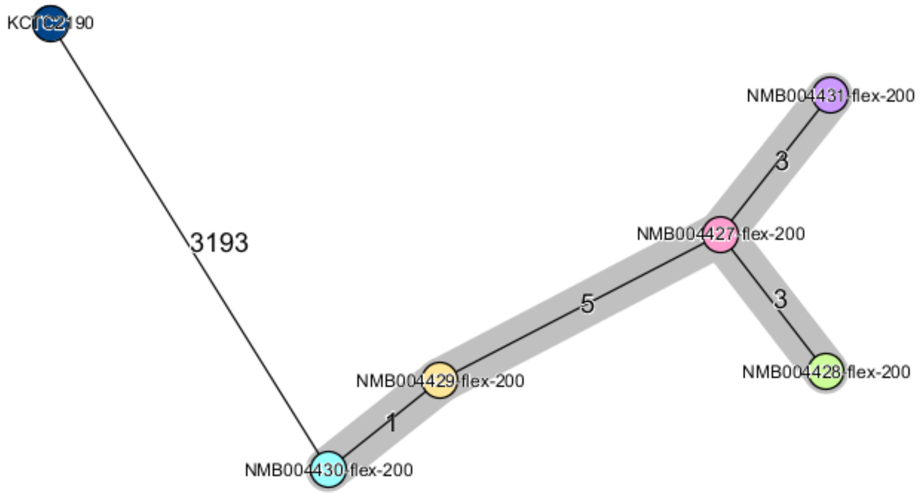

Qia-200

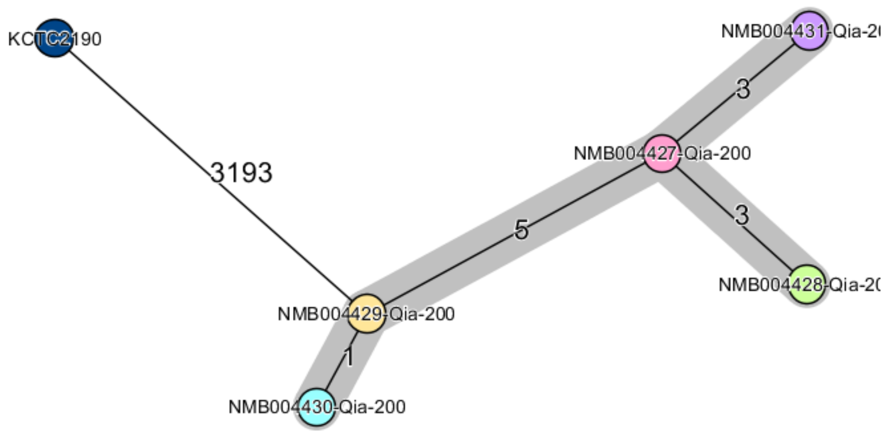

XT-100

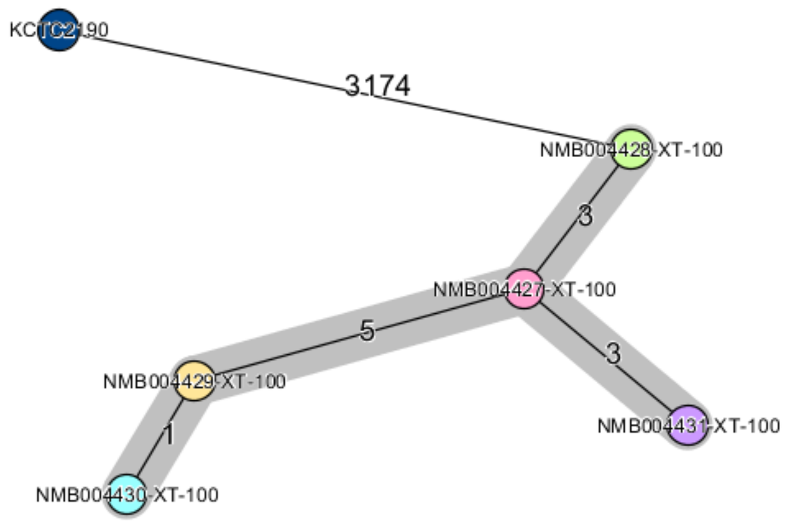

flex-100

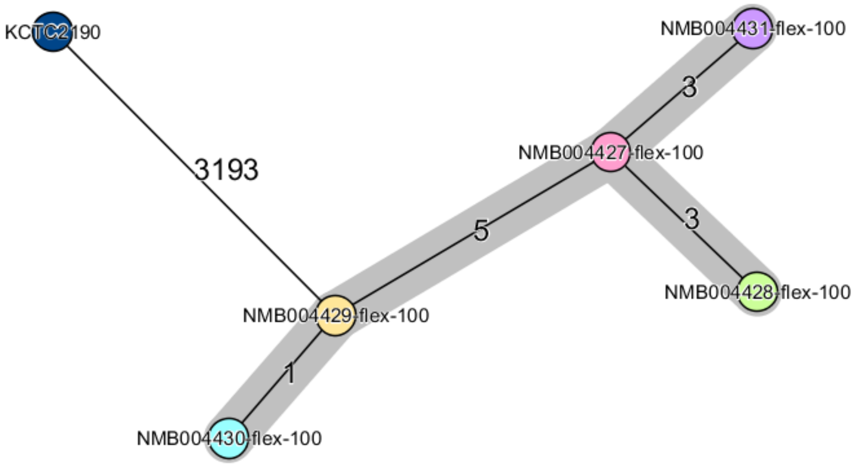

Qia-100

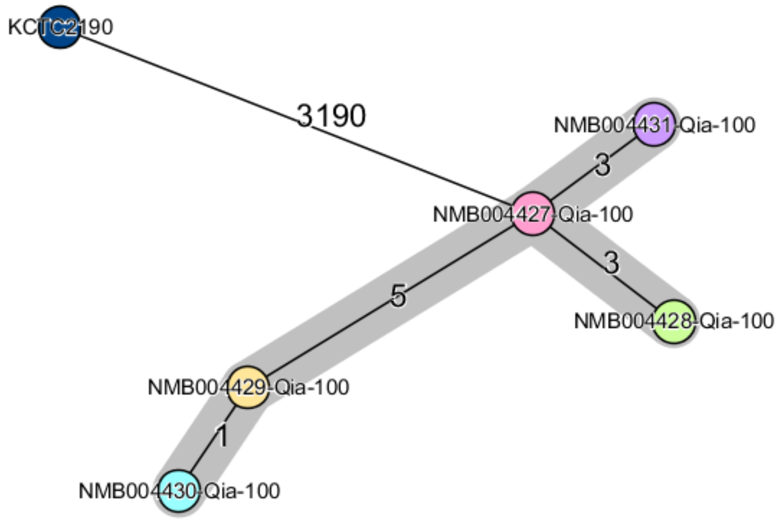

XT-50

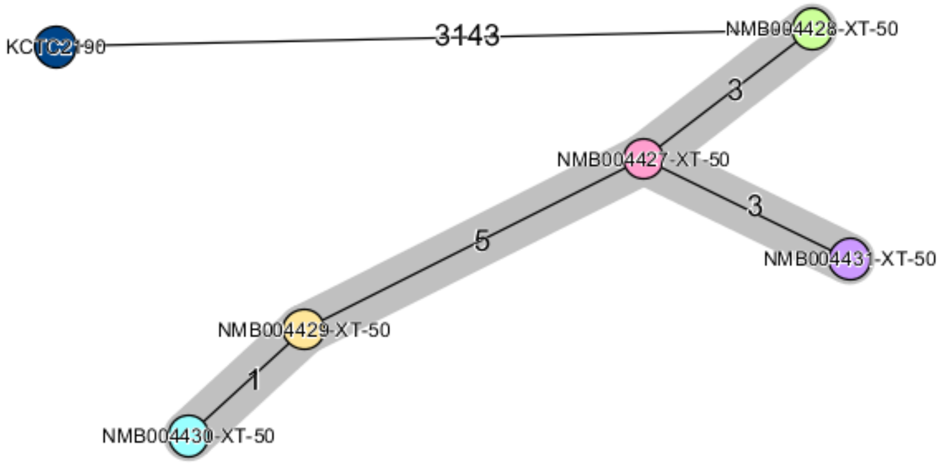

flex-50

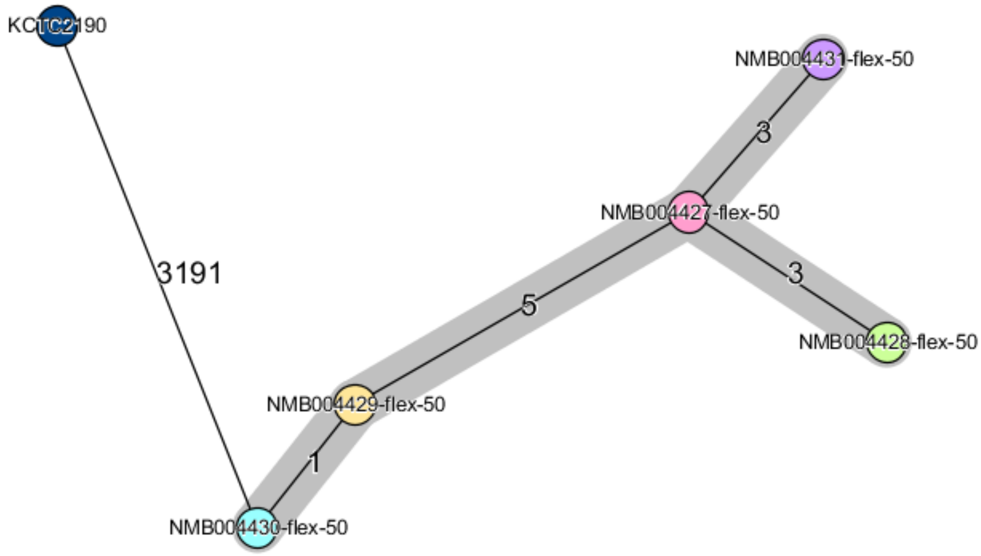

Qia-50

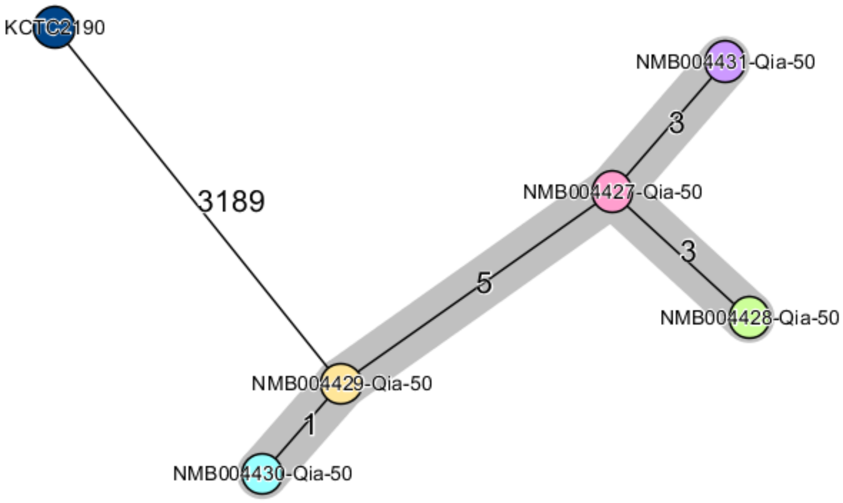

XT-20

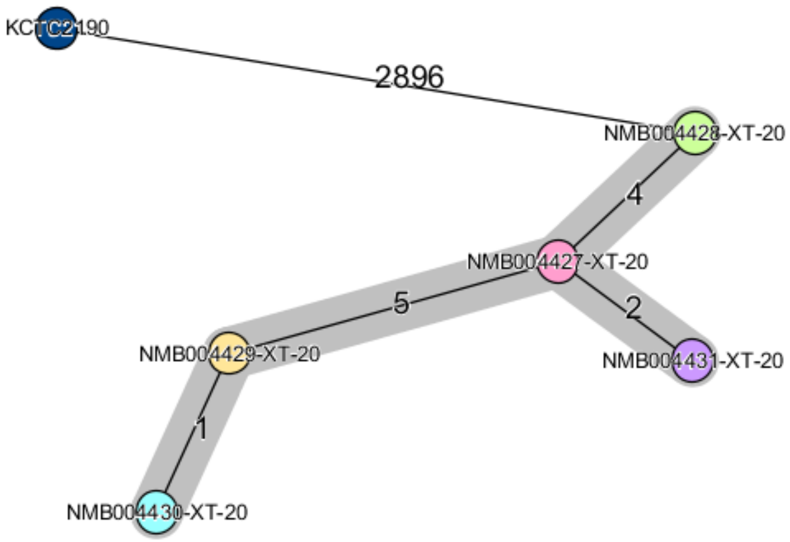

flex-20

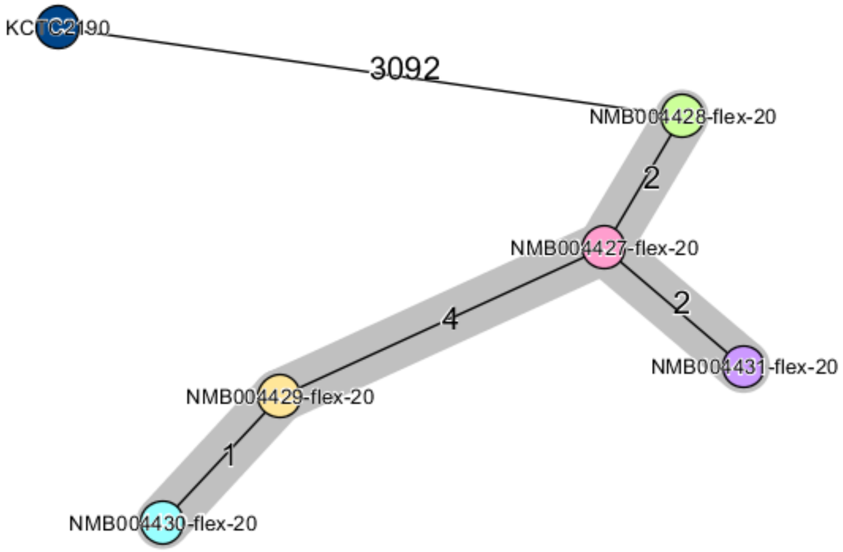

Qia-20

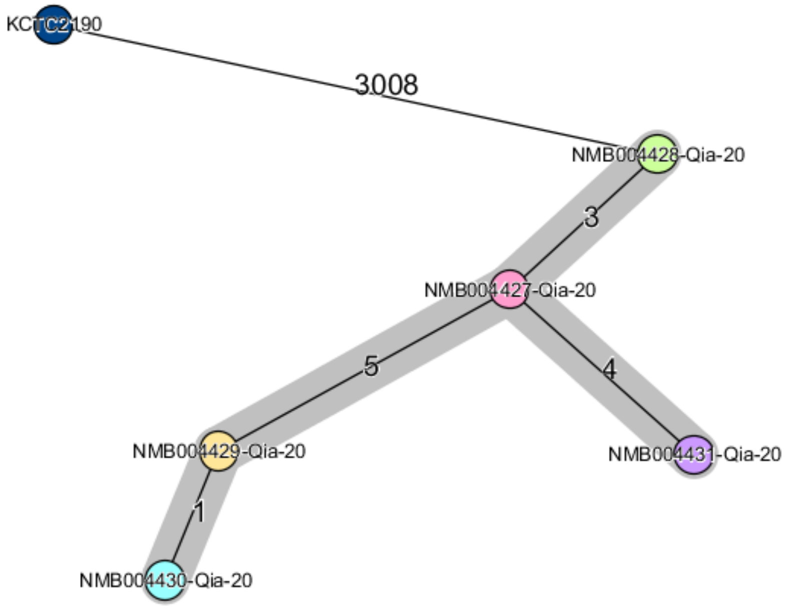

XT-10

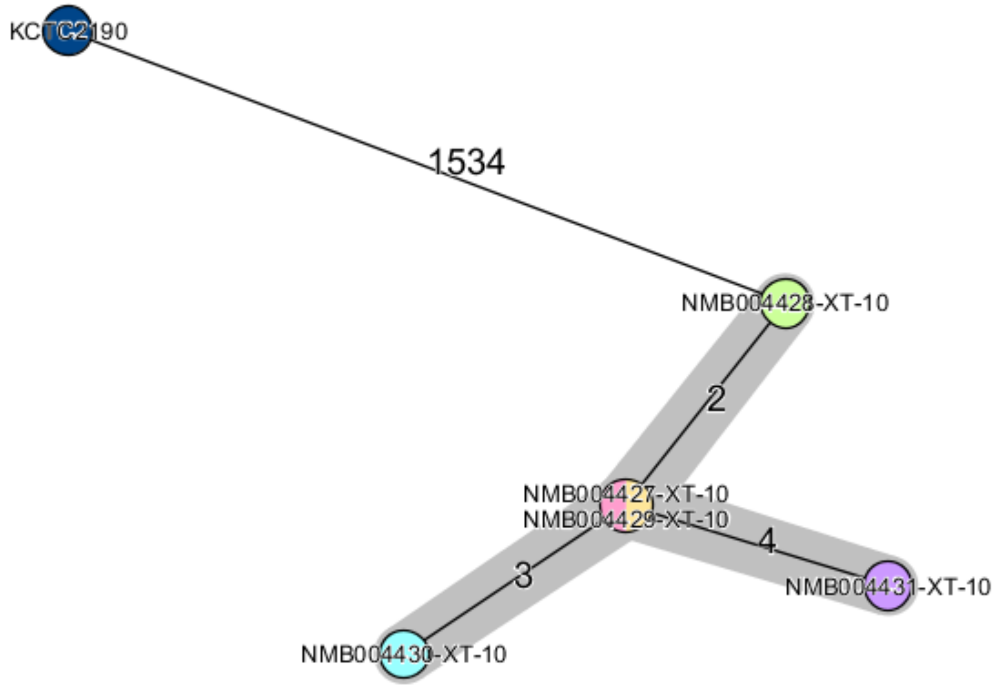

flex-10

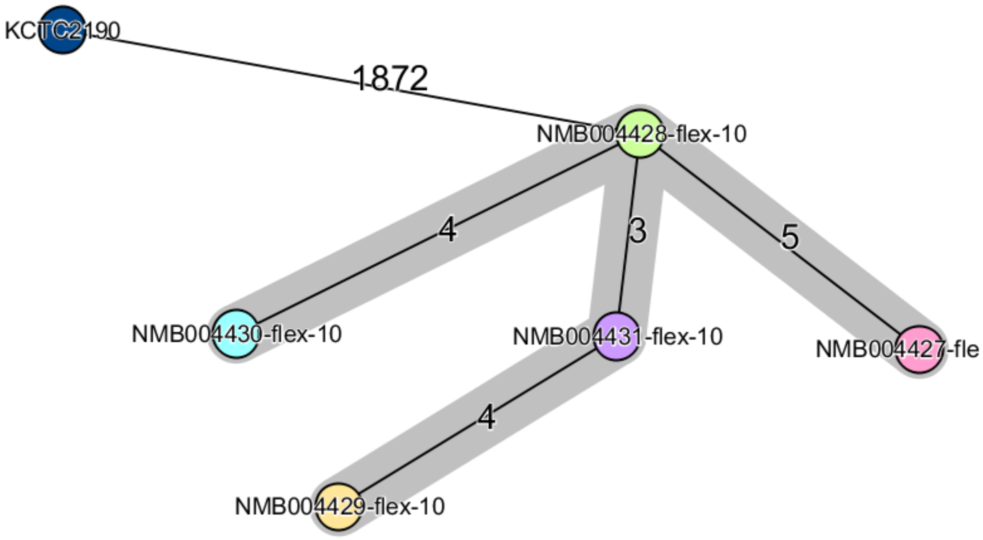

Qia-10

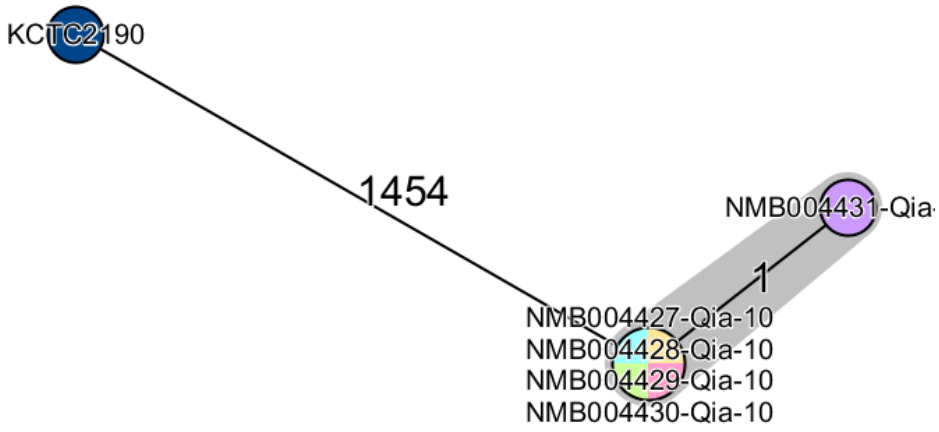

Supplement: Supplementary file 4 [file Image_4.pdf]
